# In vivo evaluation of mutant selection window of cefquinome against *Escherichia coli* in piglet tissue-cage model

**DOI:** 10.1186/s12917-014-0297-1

**Published:** 2014-12-16

**Authors:** Bingxu Zhang, Xiaoyan Gu, Yafei Li, Xiaohong Li, Mengxiao Gu, Nan Zhang, Xiangguang Shen, Huanzhong Ding

**Affiliations:** National Reference Laboratory of Veterinary Drug Residues (SCAU), College of Veterinary Medicine, South China Agricultural University, Guangzhou, 510642 China

**Keywords:** Tissue-cage models, Mutant selection window, Cefquinome, Piglets

## Abstract

**Background:**

The resistance of cephalosporins is significantly serious in veterinary clinic. In order to inhibit the bacterial resistance production, the mutant selection window (MSW) hypothesis with *Escherichia coli* (*E. coli*) ATCC 25922 exposed to cefquinome in an animal tissue-cage model was investigated.

**Results:**

Localized infection with *E. coli* was established in piglets, and the infected animals were administrated intramuscularly with various doses and intervals of cefquinome to provide antibiotic concentrations below the MIC_99_, between the MIC_99_ and the mutant prevention concentration (MPC), and above the MPC. *E. coli* lost susceptibility when drug concentrations fluctuated between the lower and upper boundaries of the window, which defined *in vitro* as the MIC_99_ (0.06 μg/mL) and the MPC (0.16 μg/mL) respectively. For PK/PD parameters, there were no mutant selection enrichment when T>MIC_99_ was ≤ 25% or T>MPC was ≥ 50% of administration interval. When T>MIC_99_ was > 25% and T>MPC was <50% of administration interval, resistance selection was observed. When AUC_24 h_/MIC_99_ and AUC_24 h_/MPC were considered, the mutant selection window extended from 32.84 h to 125.64 h and from 12.83 h to 49.09 h, respectively.

**Conclusions:**

These findings demonstrate that the MSW exists *in vivo* for time-dependent antimicrobial agents, and its boundaries fit well with those determined *in vitro*. Maintenance of antimicrobial concentrations above the MPC for > 50% of administration interval is a straightforward way to restrict the acquisition of resistance in this tissue cage model. This situation was achieved with daily intramuscular doses of 1 mg cefquinome/kg body weight.

## Background

The development of bacterial resistance has resulted from a variety of factors, including drug overuse and drug misuse [1], both in the environment and during therapy [2]. Even the commonly accepted treatment strategy of killing susceptible pathogens contributes to the problem by allowing selective amplification of resistant mutants during the treatment [3]. According to a proposed hypothesis, resistant mutants selectively amplify at antibiotic concentrations within the mutant selection window (MSW), drug concentrations between the boundary of MIC_99_ (inhibition of 99% of the cells) and mutant prevention concentration (MPC that inhibits growth of the least-susceptible single-step mutant subpopulation) [4].

The mutant selection window hypothesis was initially proposed using agar plate assays [5], and then explored in several *in vitro* or *in vivo* model [6-11]. However, as far as we know, the time-dependent drugs such as cephalosporin were rarely reported. Cefquinome is a fourth generation broad-spectrum cephalosporin antibiotic, which was developed solely for veterinary use and approved for the treatment of respiratory tract disease, acute mastitis and footrot in cattle, calf septicaemia, respiratory diseases in pigs and metritis-mastitis-agalactia syndrome in sow [12,13]. In order to reduce the occurring of cefquinome resistance and even the resistance gene transmission between herd and human, the study of mutant selection window is urgent which could be used as a framework for the design of dosage regimen of cefquinome therapy.

Localized infection is particularly suitable for determination the mutant selection window hypothesis in *in vivo* [4]. One feasible system is the tissue-cage infection model [4,14], which used a tissue-cage with holes on its surface implanted surgically into subcutaneous tissue of an animal. In piglets’ body, the surface of the cage becomes encapsulated by connective tissue 2-4 weeks after implantation, and the interior is filled with tissue-cage fluid. Bacterial cultures are injected into the cage and remained there until eliminated by host defenses and antimicrobial treatment.

In this study, above 10^10^ CFU of *E. coli* ATCC 25922 were injected into the tissue-cages implanted in piglets, and various doses and intervals of cefquinome were administered intramuscularly. The objective was to validate that the resistant mutants would be selected predominantly when drug concentrations maintained inside a concentration window, the boundaries of which determined by agar plate assays. It is hoped that this model would have the capability to provide a clear demonstration of the mutant selection window *in vivo* and support arguments for how antimicrobial dosage regimens adjustment could severely restrict the amplification and enrichment of resistant mutant for cefquinome.

## Methods

### Antimicrobials and chemicals

Penicillin, as a sodium salt for injection, and cefquinome for injection were purchased from Hebei Yuanzheng Pharmaceutical Co., Ltd., P.R. China. Cefquinome standard was from China Institute of Veterinary Drugs Control, Beijing, P.R. China. Acetonitrile and formic acid (chromatography grade) were from Fisher Scientific.

### MIC_99_, MIC, and MPC determination

*E. coli* strain ATCC 25922 stored at -70°C was grown in Mueller-Hinton broth or on Mueller-Hinton agar. MIC_99_ and MPC were determined as described elsewhere [15]. Briefly, for MIC_99_, bacterial cultures were grown overnight (≥10 h) in the constant temperature oscillation incubator at 37°C, 220 r/min, normal atmosphere, serially diluted, and approximately 10^6^ cells were applied to agar plates containing various concentrations of cefquinome. After incubation at 37°C for 16-18 h, bacterial colonies were counted, and the fraction relative to the bacterial inoculum was calculated. Drug concentration that inhibited growth by 99% was defined as MIC_99_. For MPC, above 10^10^ cells were applied to multiple cefquinome-containing agar plates. After incubation at 37°C for a total of 96 h and the examination of the appearance of colonies every 24 h, MPC was recorded as the lowest antibiotic concentration at which no colonies grew on an agar plate. The MIC_99_ and MPC were determined in the five independent experiments.

### Tissue-cage infection model

Healthy castrated cross-bred piglets (Duroc × Landrace × Yorkshire),weight ranging from 25 to 30 kg, were housed individually and fed antibiotic-free food twice a day. Water was available *ad libitum*. The experimental protocol was approved by the Committee on the Ethics of animals of South China Agricultural University (Approval number 2013-01; 15 March 2013).

The tissue-cages were made in-house from platinum-cured medical grade silicone tubing (Medical silicon, SF Medical; Beijing Jingcheng Chuangye Medical Instrument Co., Ltd., Beijing, P.R. China) and modified slightly from similar cages described by Sidhu et al. [16]. Briefly, the dimensions of the tissue-cages were of 65 mm length, 18 mm external diameter and 13 mm internal diameter. Each cage had 24 identical holes and each hole has a surface area of 9.6 mm^2^; the total exchange surface area was 2.3 cm^2^.

Two tissue-cages were implanted subcutaneously in each animal, one on either side of the neck approximately equidistant from the jugular vein and spinal cord under aseptic conditions. Surgical insertion was carried out under deep sedation (pentobarbital sodium) and local infiltration anaesthesia (procainamide hydrochloride injection) in piglets. After surgery, the piglets were treated with intramuscular penicillin (160 000 IU/kg) twice a day for 3-5 days to prevent infection. The non-steroidal anti-inflammatory drug (NSAID) was provided for analgesia in post-operation simultaneously. By 4 weeks after implantation, each tissue-cage had become sealed with a thin layer of connective tissue and had been filled with clear, yellowish tissue-cage fluid. Above 10^10^ CFU of exponentially growing *E. coli* ATCC 25922 culture was concentrated in 1 mL of saline and injected into each tissue-cage. Two days after infection, 0.5 mL of tissue-cage fluid was withdrawn from each cage for a viable-bacteria count. Piglets having above 10^8^ CFU/mL viable bacterial cells in tissue-cage fluid were treated with various doses and intervals of cefquinome.

### Pharmacokinetic measurements

Eighteen piglets were randomly allocated to 7 administration groups and treated at 0.1, 0.2, 0.4, 0.8, or 1.0 mg/kg of body weight once a day (24 h interval) or 0.2 and 0.4 mg/kg of body weight twice a day (12 h interval). 0.1, 0.8, 1.0 mg/kg groups had 2 piglets and 4 tissue cages of each group. 0.2, 0.4 mg/kg (12 h and 24 h interval) groups had 3 piglets and 6 tissue cages of each group, which had one more piglet compared to 0.1, 0.8, 1.0 mg/kg groups respectively because these two dosages easily induced resistant mutation. And this series of dosages were determined by recommended dose which was 2 mg cefquinome/kg body weight in intramuscularly once daily for 3-5 days [13] and pre-experiments data (not provided). Cefquinome were administrated intramuscularly (intragluteal muscles) for consecutive 5 times beginning on the 3^rd^ day after infection with *E. coli* ATCC 25922 for every piglet in administration groups. The control group, three piglets, received sterile physiological saline (1 mL) simultaneously in the same way. Tissue-cage fluid (0.5 mL) was collected from the cage at 1, 3, 6, 9, 12, and 24 h after each administration in group with 24 h interval. For groups with 12 h interval, samples were collected at 1, 3, 6, 9, 12 h after each administration. Fluid samples were clarified by centrifugation at 3 000 × g for 10 min and stored at -20°C.

The concentrations of cefquinome was determined using an Agilent 1200 series high performance liquid chromatography and an Agilent 6400 triple quadrupole mass spectrometer equipped with an electrospray ionization source (HPLC-MS/MS, Agilent Technologies, USA). The chromatographic separation was achieved on a Phenonenex BDS C_18_ column (150 mm × 2 mm; internal diameter, 5 μm, Phenomenex Technologies) at 40°C with a thermostat column oven (Agilent 1200 series, Agilent Technologies). The mobile phase consisted of solution A (water with 0.1% formic acid, V/V) and solution B (acetonitrile) at 0.25 mL/min flow rate. The gradient elution was: 0-1 min, 5% B; 1-5.5 min, 60% B; 5.5-10 min, 5% B. The injection volume was 5 μL.

A calibrated curve was constructed by adding a known amount of cefquinome to blank tissue-cage fluid over concentrations ranged from 0.001 μg/mL to 1 μg/mL. The lower limit of quantification (LLOQ) of cefquinome was 5 ng/mL. The recoveries of cefquinome in tissue-cage fluid were 94.2 ± 7.34% (mean ± standard deviation, SD, *n* = 5). The coefficients of variability (CV%) were all < 10% for both intra-assay and inter-assay variation.

Pharmacokinetic/pharmacodynamic (PK/PD) indices such as T>MIC_99_, T>MPC, AUC/MIC_99_, AUC/MPC, C_max_/MIC_99_, C_max_/MPC were calculated according to a noncompartmental analysis using WinNonlin programme (version 6.1, Pharsight Corporation, Mountain View, CA, USA). The liner trapezoidal rule was used to calculate the area under the concentration-time curve (AUC). All PK/PD indices calculations referred to the 12 h and 24 h dosing interval immediately following the fifth injection after finishing administration using the cefquinome concentrations in tissue cage fluid.

### Loss of susceptibility to cefquinome

Potential loss of susceptibility was monitored in tissue-cage fluid (0.5 mL/cage) obtained daily before and during the cefquinome treatment (after every administration) and 24 and 48 h after the termination of treatment. To amplify cultures, half of each sample was incubated overnight in drug-free Mueller-Hinton broth, and then the MIC was determined with the CLSI [17] agar dilution method. The other half of each sample was serially diluted with sterile physiological saline and applied to agar either lacking drug or containing cefquinome at 1 × MIC of the starting culture. After incubation at 37°C for 24-48 h, colonies were calculated, and the fraction of mutants in the population was calculated.

Resistant mutants (growing on 1 × the MIC of cefquinome-containing agar) were also chosen randomly from samples that had cefquinome concentrations predominantly in the lower, middle, or upper part of the selection window with 12 h or 24 h interval administration. Single colonies of these mutants were passaged 5 times on drug-free agar, and the MIC to cefquinome was then determined.

### Statistical analysis

Fisher’s exact test was used for statistical analysis of the PK/PD data, with an infected but untreated set of piglets (3 piglets, 6 tissue-cages) as a control. P < 0.05 was considered to be statistically significant.

## Results

### Bacteria count in tissue-cage model

Two perforated tissue-cages were implanted into each piglet. When above 10^10^ CFU bacteria were injected into an implanted cage, no severe illness or distress occurred during a 10-day observation. Bacterial concentrations remained constant at about 10^8^ CFU/mL when piglets were treated intramuscularly for 5 times with sterile physiological saline once or twice daily.

**Figure 1:**
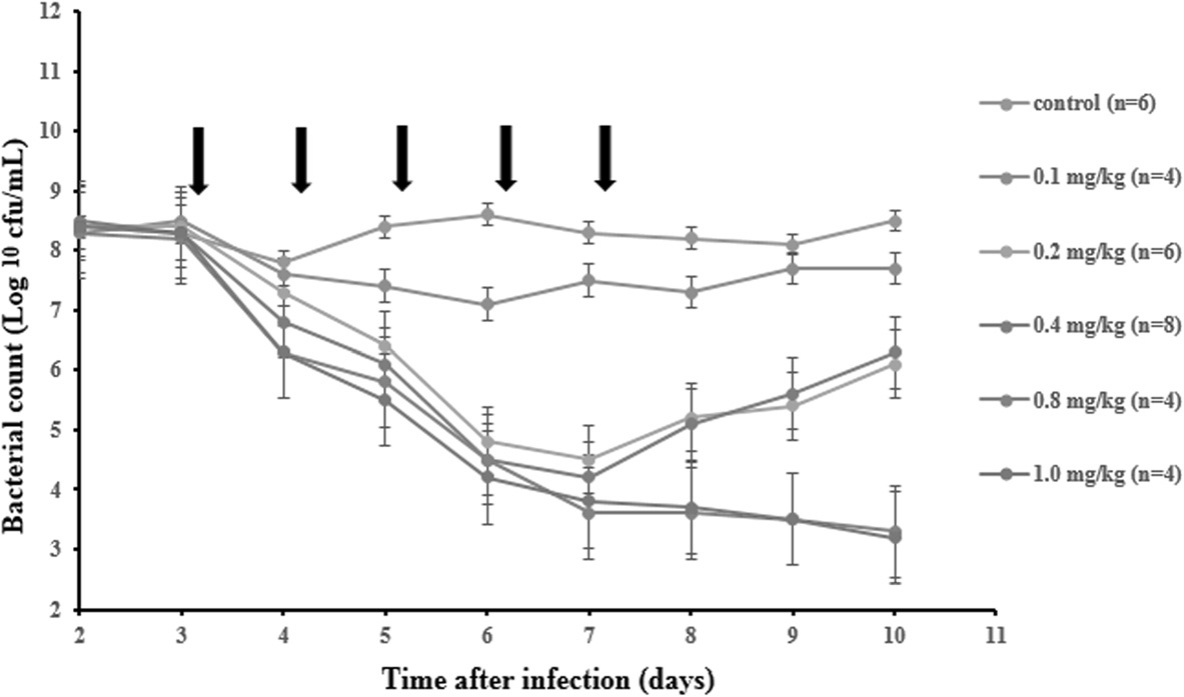
**Effect of cefquinome dose on bacterial inhibition in the tissue-cage model.** Tissue-cage implantation and *Escherichia coli* ATCC 25922 infection were done as described in Materials and Methods. Three days after infection, various doses (0, 0.1, 0.2, 0.4, 0.8, or 1.0 mg/kg of body weight, each piglet has tissue-cages) of cefquinome were administered intramuscular once daily for 5 days (indicated by the arrow). Bacterial colony-forming units in tissue-cage fluid was monitored at 24 h intervals beginning 1 day before the initiation of cefquinome in treatment and ending 2 days after the termination of cefquinome treatment.

Administration of cefquinome at 0.1 mg/kg at 24 h interval slightly reduced bacterial numbers compared to the control during the trial. Administration of cefquinome at 0.2 and 0.4 mg/kg at 24 h interval observably reduced bacterial numbers for the first 4 administrations, but bacterial growth was observed later during treatment and during post-treatment. Administration of cefquinome at 0.8 and 1.0 mg/kg at 24 h interval caused bacterial numbers to decrease throughout treatment and remain low during the growth recovery phase (Figure 1).

### Cefquinome concentrations

**Figure 2:**
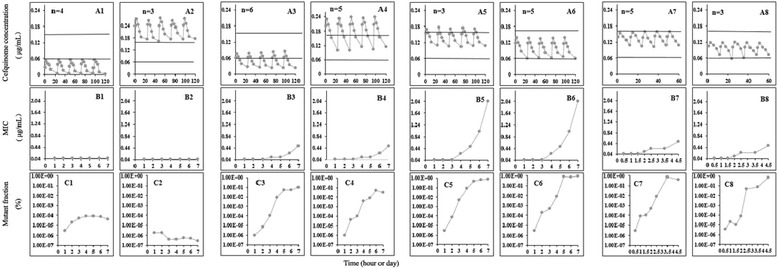
**Effect of actual cefquinome concentration on loss of susceptibility and mutant enrichment.** Tissue-cage implantation and *Escherichia coli* ATCC 25922 infection were done as described in Meterials and Methods. Piglets having above 1 × 10^8^ CFU/mL *E.coli* in tissue-cage fluid 2 days after infection were treated with various intramuscular doses of cefquinome once daily and twice daily for 5 times beginning 3 days after infection. The dosage, administration at hour or day 0 of the x-axis, protocol for each panel was as follows: **A1**, 4 received 0.1 mg/kg; **A2**, 3 received 1.0 mg/kg; **A3**, 4 received 0.2 mg/kg and 2 received 0.4 mg/kg; **A4**, 4 received 0.8 mg/kg and 1 received 1.0 mg/kg; **A5**, 3 received 0.4 mg/kg; **A6**, 2 received 0.2 mg/kg and 3 received 0.4 mg/kg; **A7**, 1 received 0.2 mg/kg and 4 received 0.4 mg/kg; **A8**, 3 received 0.2 mg/kg. Cefquinome concentration in tissue-cage fluid was monitored at the indicated times after the administration of each dose **(panels A1-A8)**. The boundaries of the mutant selection window (the MIC_99_ and MPC) were determined with the *E.coli* inoculum by agar plate assays. Tissue-cage fluid was sampled for bacteria at 24 h and 12 h intervals for 7 times starting immediately before the administration of the first dose of cefquinome. Loss of susceptibility **(panels B1-B8)** was monitored as an increase in MIC averaged for all piglets in the group. The fraction of resistant mutants **(panels C1-C8)** in each group of piglets was determined daily as the number of colonies grown on cefquinome-containing agar (MIC) relative to the number that grew on drug-free agar.

The values of MIC, MIC_99_, and MPC were 0.064 μg/mL, 0.06 μg/mL, and 0.16 μg/mL in present study, respectively. Cefquinome concentrations, determined in samples of tissue-cage fluid collected at various time points over several days, are shown in Figure 2 (panels A1-A8). There were totally 34 tissue cages analyzed and another 2 tissue cages were excluded because of bacterial pollution. And the cefquinome concentrations shown for each of the 8 groups were the means of the concentrations from all animals/tissue cage fluids selected for those groups. The boundaries of the mutant selection window were determined to be 0.06 μg/mL (MIC_99_) and 0.16 μg/mL (MPC) by agar plate assays, which were the average values determined in the five independent experiments. There are 8 groups (A1-A8) to display the different classifications (lower, higher, partially inside, totally inside) of cefquinome concentrations based on the MSW boundary.

### MICs and mutant fraction

Samples of *E. coli* ATCC 25922 in tissue-cage fluid after treatment with various doses were examined for susceptibility to cefquinome. Increases in MICs were readily observed (Figure 2, panels B3-B8) when cefquinome concentrations were partially or totally inside the selection window (Figure 2, panels A3-A8).

When cefquinome concentrations maintained either below the MIC_99_ (Figure 2, panel A1) or above the MPC (Figure 2, panel A2), no MIC increase, either during (days 0-5) or after therapy (days 6-7), was observed (Figure 2, panels B1 and B2). When cefquinome concentrations overlapped the lower window boundary (MIC_99_) and the C_max_ was lower than the middle concentration (0.11 μg/mL) of the MSW during treatment (Figure 2, panel A3), decreased susceptibility (MIC increase) was detected in 1 of 6 cages and the MIC increased to 0.512 μg/mL at day 7 (Figure 2, panel B3). When cefquinome concentrations crossed the upper boundary of the window (MPC) and the C_min_ was higher than the middle concentration of the MSW during treatment (Figure 2, panel A4), loss of susceptibility was detected in 1 of 5 cages and the MIC also increased to 0.512 μg/mL at day 7 (Figure 2, panel B4). As drug concentrations were over the MIC_99_ and T>MPC did not exceed 25% of interval, susceptibility decreased in 8 of 8 cages and the MIC increased to 2.048 μg/mL at day 7 (Figure 2, panels B5 and B6), regardless of the mean concentration located into the lower (Figure 2, panel A6) or upper (Figure 2, panel A5) part of the selection window. In addition, cefquinome susceptibility decreased in all 8 cages and the MIC increased to 0.512 μg/mL at day 5 (Figure 2, panels B7 and B8) in the condition of 12 h interval with drug concentrations approximately the same with the condition of 24 h interval (Figure 2, panels A5 and A6). The detailed information is shown in Table 1.

**Table 1:** **Values of PK/PD parameters and change of MIC in different cefquinome concentrations (18 piglets, 7 groups, treated at 0.1, 0.2, 0.4, 0.8, or 1.0 mg/kg of body weight once a day or 0.2 and 0.4 mg/kg of body weight twice a day)**

**Groups**	**PK/PD parameters**	**Interval (h)**	**MIC (μg/mL)**
A1 (N = 2)	T > MIC_99_% = 0	24	Total 4 cages, no increase
A2 (N = 2)	T > MPC% = 100%	24	Total 3 cages, no increase
A3 (N = 3)	T > MIC_99_% = 25%	24	1/total 6 cages, increased to 0.512
	T > MPC% = 0		
	T_MSW_% = 25%		
A4 (N = 3)	T > MIC_99_% = 100%	24	1/total 5 cages, increased to 0.512
	T > MPC% = 50%		
	T_MSW_% = 50%		
A5 (N = 2) & A6 (N = 3)	T > MIC_99_% = 100%	24	8/total 8 cages, increased to 2.048
	T > MPC% ≤ 25%		
	75% ≤ T_MSW_% ≤ 100%		
A7 (N = 3) & A8 (N = 2)	T > MIC_99_% = 100%	12	8/total 8cages, increased to 0.512
	T > MPC% = 0		
	T_MSW_% = 100%		

Cefquinome PK/PD values were determined from tissue cage fluids using total drug concentrations. N is the animal number per group.

The fraction of mutants dramatically increased (>10^4^ fold) when drug concentrations fell inside the selection window. And there was no difference no matter whether the concentration partly or wholly dropped into the MSW (Figure 2, panels C3-C8). Overall, these data have shown that the selection window boundaries determined by agar plate assays fit well with results obtained *in vivo* in piglets for cefquinome.

### Correlation of PK/PD indices with mutant enrichment and amplification

PK/PD indices, such as AUC_24 h_/MIC_99_ (where AUC_24 h_ is the area under the drug concentration time curve in a 24 h interval) and time above the MIC_99_, provide an empirical way to relate antimicrobial dose to favorable treatment effect for bactericidal agents. Relationships between PK/PD indices, determined as steady-state values after the fifth dose, and lost of susceptibility are shown in Table 2. For cephalosporin, T>MIC_99_ is the index most commonly associated with restricting susceptible cell growth. Only 1 of 10 tissue-cages lost susceptibility when T>MIC_99_≤6 h or T>MIC_99_%≤25% (Table 2 and Figure 2, panels A1 and A3). T>MPC is probably the appropriate parameter for the upper boundary of the selection window. Only 1 of 8 tissue-cages was lost susceptibility when T>MPC≥12 h or T>MPC%≥50% (Table 2 and Figure 2, panels A2 and A4). Loss of bacterial susceptibility occurred in 16 of 16 tissue-cages (8 for 24 h interval and 8 for 12 h interval) when T_MSW_% was between 25% and 100%, with T>MPC%≤25% simultaneously (Table 2 and Figure 2, panels A5-A8).

**Table 2:** **Correlation of pharmacokinetic/pharmacodynamics (PK/PD) parameters with selection of resistance**

**PK/PD index, value** ^**a**^	**Fraction of tissue-cages with resistant bacteria (mutant/total)**	**P** ^**b**^
T > MIC_99_		
≤25%	1/10	0.625
>25%	17/24	0.003
T > MPC		
<50%	17/26	0.006
≥50%	1/8	0.400
AUC_24 h_/MIC_99_ (h)		
≤32.84	1/8	0.571
32.84-125.64	16/20	0.001
≥125.64	1/6	0.500
AUC_24 h_/MPC (h)		
≤12.83	1/8	0.571
12.83-49.09	16/20	0.001
>49.09	1/6	0.500
C_max_/MIC_99_		
≤1.32	1/9	0.600
1.32-4.26	16/18	0.0002
≥4.26	1/7	0.539
C_max_/MPC		
≤0.52	1/9	0.600
0.52-1.66	16/18	0.0002
≥1.66	1/7	0.539

All PK/PD parameters were determined using total drug concentrations in tissue cage fluid. Total 34 tissue cages were analyzed and 2 tissue cages excluded because of bacterial pollution.

^a^Tine in the window (T_msw_) is not presented in the table because it fell into 2 categories.

^b^P values were calculated by Fisher^’^s exact test, with a set of 3 infected but untreated piglets (6 tissue-cages) used as a control. High values indicate no difference with the control.

Other PK/PD indices also showed statistically significant (P < 0.05) correlations with the selection of resistance (Table 2). When AUC_24 h_/MIC_99_ and AUC_24 h_/MPC were considered, the mutant selection window extended from 32.84 h to 125.64 h and from 12.83 h to 49.09 h, respectively. In another example, the selection window extended from 1.32 to 4.26 and from 0.52 to 1.66, respectively, when maximum concentration C_max_/MIC_99_ and C_max_/MPC were considered.

## Discussion

Antimicrobial resistance becomes an increasingly serious problem that is likely to require attention at many levels [2]. Issues concerning dosing are addressed by the mutant selection window hypothesis [18]. The fundamental difference between the traditional MIC-based strategies and the MPC-based approach is that the former requires bacteria to acquire only 1 mutation for growth in the presence of drug, whereas the latter requires 2 or more [19].

Considering the boundaries of the selection window, it need to be predictable on the basis of data obtained by clinical microbiological laboratories. Previous work has shown that static agar plate values of MIC_99_ and MPC of fluoroquinolones fit well with selection window boundaries obtained in *in vitro* models [4,6,8,20]. The data in Figure 2 demonstrate that agar plate determinations of MIC_99_ and MPC fit well with the boundaries of the selection window seen *in vivo* at the site of infection for cefquinome.

In PK/PD model, the T>MIC (for time-dependent drugs) and AUC_24 h_/MIC (for concentration-dependent drugs) can be used empirically to predict favorable effect when susceptible populations are considered [21]. Herein, the T>MIC_99_ (for time-dependent drugs) or AUC_24 h_/MIC_99_ (for concentration-dependent drugs) can be used to define the lower boundary of the selection window in PK/PD combined with MSW model. For the upper boundary of the window, as the MPC is the MIC of the least-susceptible single-step mutant, T>MPC or AUC_24 h_/MPC probably is the appropriate parameter [22]. An *in vitro* study of *E. coli* treated with ciprofloxacin also argued for the use of AUC_24 h_/MPC. Other PK/PD indices—such as C_max_/MIC_99_, C_max_/MPC, and time in the window (T_MSW_ %)—also showed a statistically significant (P < 0.05) correlation with the selection of resistance.

In present study, a correction is required when concentrations are high enough to kill the resistant (the cefquinome concentration needed to exceed the MPC for over half the dosing period to restrict the recovery of mutants). Keeping antimicrobial concentrations above the MPC >12 h (T>MPC% >50%) or AUC_24 h_/MPC >49.09 h is a straightforward way to restrict the acquisition of resistance in this study. However, it is still complicated that what concentrations made the susceptible strains acquired resistance inside the window. In previous investigation, concentrations at the center of the window were suitable for selecting a double mutant in an *in vitro* model [23]. In present piglet/perforated-tissue-cage system, the drug concentration needed to be inside the window for ≥75% of the interval for enrichment mutants when those concentrations fluctuated above and below the MPC. When the concentrations fluctuated above and below the MIC_99_, they needed to be inside the window for only 25% of the interval. This difference probably results from: (1) more abundant preexisting resistant mutant subpopulations being able to survive and propagate near the bottom of the window; (2) the killing of some mutants when drug concentrations are close to the top of the window; (3) the organism have a better growth fitness at a low concentration than a high condition [22].

**Figure 3:**
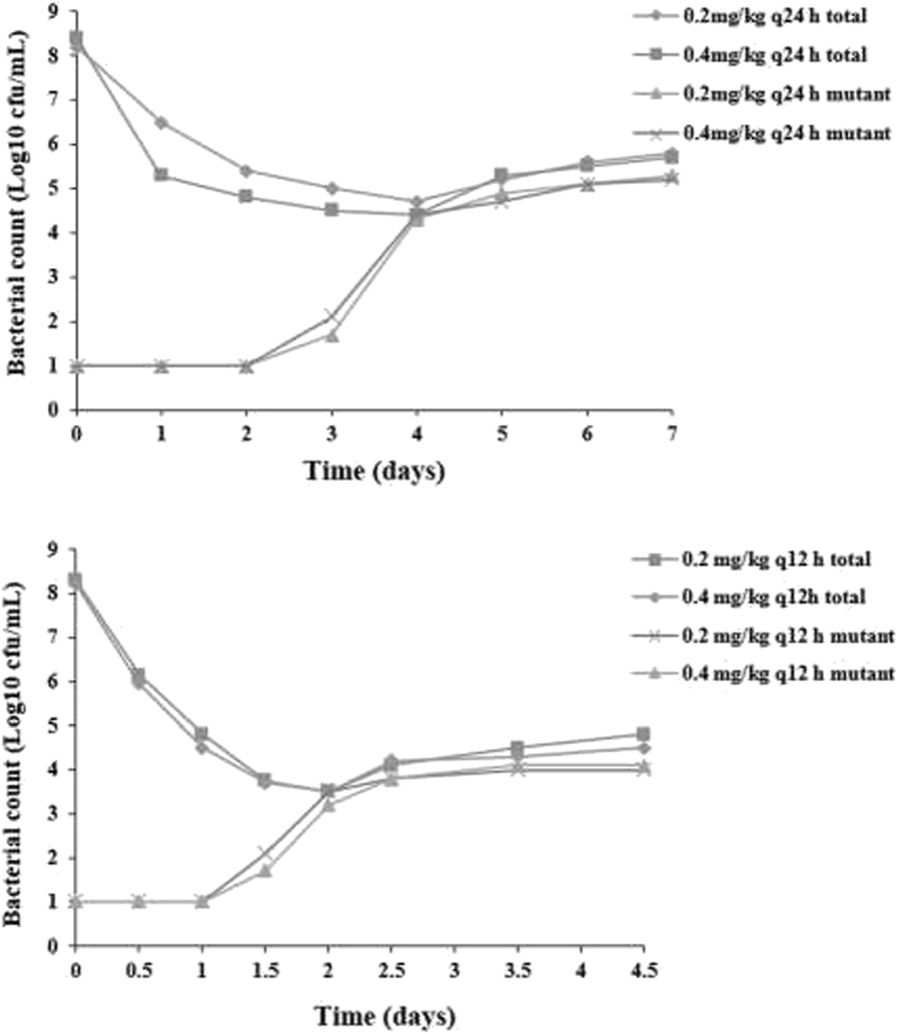
**Effect of cefquinome exposure on recovery of total and resistant bacteria.** Concentrations of total bacteria and resistant mutants were determined in aliquots of tissue-cage fluid obtained at the indicated time points after the initiation of treatment. Representative two examples (one piglet each dose regimen) are shown for piglets in which the cefquinome concentration was inside the mutant selection window, as determined in Figure 2.

The fraction of mutants can increase either by mutant amplification (outgrowth of mutant cells) or by mutant enrichment (killing of susceptible cells). To distinguish these situation, we determined the absolute number both of total and resistant bacteria. When drug concentrations were inside the selection window, the total population size decreased and then gradually increased. However, the mutant numbers were initially constant, indicating that a fractional increase may result from preferential killing of susceptible cells. After several times of administration, amplification of mutants was observed at last (Figure 3). Thus, the selection of cefquinome-resistant mutants *in vivo* probably arose from both mutant amplification (outgrowth of mutant cells) and mutant enrichment (killing of susceptible cells).

Beta-lactam antibiotics, including penicillins, cephalosporins and carbapenems, are widely used not only in human but also in vet clinic. Production of beta-lactamase is one of major resistance mechanisms in gram-negative bacteria, which mostly refers to extended-spectrum beta-lactamases (ESBLs) existing largely in microorganisms of the family Enterobacteriaceae [24]. It was complicated to confirm the mechanism of acquisition resistant genes for sensitive strains exposure of antimicrobial agents, such as chromosome gene mutant and plasmid acquisition. The present data could not display how the resistant property was produced by *E. coli* ATCC 25922. More molecular technique, the PCR of target gene, the PFGE of original strain and mutants, and plasmid profiles analysis at least, should be applied to determine the mutant gene which caused bacterial resistance. Those are the mainly study purposes of the next task in laboratory animals.

## Conclusions

In conclusion, the data in Figure 2 demonstrate that the mutant selection window agar plate determinations fit well with the piglets infected with *E. coli* ATCC 25922 of cefquinome treatment *in vivo*. Because agar plate assays are routine in clinical laboratories, implementation of selection window dosing strategy is feasible. The next steps for the tissue-cage model are to obtain more data to confirm the boundary of MSW. For example, more virulent strains should be used to allow bacterial populations to reach 10^10^ cells by *in vivo* growth from a smaller inoculum in further study.

## Abbreviations

MSW: Mutant selection window
*E.coli*: *Escherichia coli*
MIC: Minimum inhibitory concentration
MPC: Mutant prevention concentration
PK: Pharmacokinetics
PD: Pharmacodynamics
AUC: Area under the curve
CFU: Colony forming unit
ATCC: American type culture collection
NSAID: Non-steroidal anti-inflammatory drug
HPLC: High performance liquid chromatography
MS: Mass spectrometry
LLOQ: Lower limit of quantification
CV: Coefficients of variability
CLSI: Clinical and laboratory standards institute
ESBLs: Extended-spectrum beta-lactamases
PCR: Polymerase chain reaction
PFGE: Pulsed field gel electrophoresis
